# High-Performance Photocatalytic H_2_ Production Using a Binary Cu/TiO_2_/SrTiO_3_ Heterojunction

**DOI:** 10.1021/acsaem.3c00219

**Published:** 2023-03-23

**Authors:** Marcos González-Tejero, Joyce G. Villachica-Llamosas, Alba Ruiz-Aguirre, Gerardo Colón

**Affiliations:** † Instituto de Ciencia de Materiales de Sevilla, Centro Mixto Universidad de Sevilla-CSIC, Américo Vespucio, 49, 41092 Sevilla, Spain; ‡ CIEMAT—Plataforma Solar de Almería, Ctra. De Senés s/n., 04200 Tabernas, Almería, Spain

**Keywords:** photocatalysis, hydrogen, strontium titanate, TiO_2_, heterojunction

## Abstract

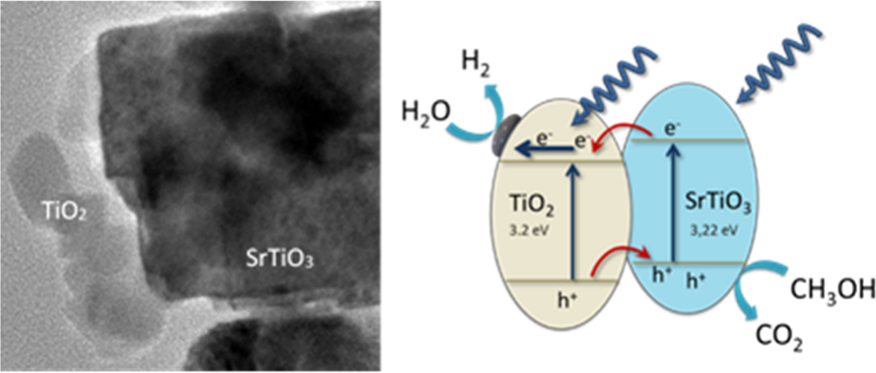

Cu/TiO_2_/SrTiO_3_ hybrid structures have been synthesized by the simple impregnation method from Cu/TiO_2_ and SrTiO_3_ systems. The structural and surface characterization stated that Cu/TiO_2_/SrTiO_3_ composites form an effective covering of SrTiO_3_ by Cu/TiO_2_. The heterostructured catalysts lead to an outstanding improved photoactivity for hydrogen production from methanol photoreforming that would be related with the efficient separation of charge pairs favored by the Cu/TiO_2_/SrTiO_3_ heterojunction. The best photoproduction is attained for the 30 wt % SrTiO_3_ heterojunction showing 81.7 mmol/g H_2_ after 6 h (leading to an apparent quantum yield of ca 1%), 1.7 times higher than that of bare Cu/TiO_2_.

## 1 Introduction

Over the last few years, the energy scenario has become progressively more complex.^1^ Although ending the dependence on fossil fuels is a real challenge, the search for alternative sources is urgently needed. Within this frame, hydrogen is considered the ideal clean and sustainable alternative to the actual scheme. As a result, hydrogen production from sustainable processes has attracted much interest. For many years, the photocatalytic splitting of water toward hydrogen evolution has constituted the holy grail to a green energy production.^2^ Moreover, the possibility of using solar energy would allow a large-scale utilization of this technology in the future.

In this respect, despite enormous efforts to generate hydrogen through powder-based solar water splitting systems, the efficiency values required for practical applications are unfortunately rather modest to date.^3,4^

It has been widely reported that H_2_ production from the alcohol photoreforming reaction appears as a more feasible alternative to water splitting.^5−8^ The main drawbacks for the photocatalytic H_2_ production are based on different factors concerning the catalyst performance such as the occurrence of the backward reaction, the rapid recombination of photogenerated carriers, or even the deactivation of the catalyst, which hindered the development of H_2_ production at a large scale.^9,10^ The improvement of the photocatalytic efficiency by inducing the separation of photogenerated charges has been largely studied. Within the different approaches, the use of metal co-catalysts as charge trapping sites has turned necessary for enhancing the efficiency of the photocatalytic reaction by avoiding the electron–hole recombination processes.^11−13^

By following this strategy, two important effects can be achieved. First, the increase of the separation efficiency of the photogenerated pairs during the photocatalytic mechanism, and second considering narrower-band-gap semiconductors that would extend the absorption range of the photoactive system. Along these lines, it has been argued that recent advances in the tailoring of new photocatalysts for solar applications might allow the understanding of the band electronic structure that would lead to an effective handling of charge carriers.^14^ Tailored heterostructures such as TiO_2_/BiPO_4_ demonstrated that the combination of semiconductors with the adequate band disposition would exhibit an improved photoactivity with respect to that of the single semiconductors.^15^ SrTiO_3_ is a photoactive semiconductor that has also shown interesting performance for H_2_ production.^16−19^ The reported band structure for SrTiO_3_ clearly allows the suitable cooperative junction with TiO_2_, forming a staggered coupling of bands.^20,21^ Moreover, due to the nearly epitaxial matching showed by these two semiconductors, a favorable lattice mismatch between the (001) surfaces of the two semiconductors would minimize the strain in the heterostructure.^14,22^ On this basis, through tailored band gap engineering, the heterostructuration of TiO_2_ and SrTiO_3_ semiconductors has reported interesting synergies.^23,24^

As mentioned above, the presence of metal co-catalysts has been demonstrated to be necessary for enhancing the photonic efficiency of the photocatalytic process by avoiding the electron–hole recombination processes.^25−27^ The addition of noble metals has different effects on the photoactivity. Such an effect is obviously affected not only by the nature of the metal but also by other parameters such as sample history and metal features.^28^ Noble metals have been reported to show higher performances. However, as a cheaper alternative, copper-based catalysts have also been extensively considered.^6,29−32^

Therefore, the combination of a cheap co-catalyst with a tailored band gap strategy would be an interesting approach that would enable the scaling up of hydrogen production.

## 2 Experimental Section

### 2.1 Catalyst Preparation

#### 2.1.1 SrTiO_3_

Strontium titanate was synthesized by a microwave-assisted hydrothermal method. First, 3.3 mL of titanium isopropoxide was added to 50 mL of NaOH (1 M) in ethanol containing the stoichiometric amount of Sr(NO_3_)_2_ under vigorous stirring. The white slurry was enclosed in a Teflon vessel and heated at 200 °C during 1 h. The obtained precipitate was cooled until room temperature, filtered, washed repeatedly, and finally dried overnight at 90 °C. The obtained systems were denoted as STO.

#### 2.1.2 Cu/TiO_2_

Copper was deposited by the chemical reduction method. Thus, 1 g of commercial TiO_2_ (Evonik P25) was suspended in 100 mL of water containing the stoichiometric amount of the copper nitrate precursor to achieve 2 wt % of metal loading. Metal deposition was achieved by chemical reduction using NaBH_4_ as a reducing agent for 30 min under stirring at room temperature. The obtained systems were filtered, thoroughly washed with distilled water, and dried at 90 °C. Cu-doped systems were denoted as CuP25.

#### 2.1.3 Cu/TiO_2_/STO

Heterostructured composites were obtained by the simple impregnation method.^33,34^ In a typical procedure, the corresponding amounts of STO and CuP25 were suspended in ethanol solution. Both suspensions were sonicated for 15 min before mixing. The final suspension containing both semiconductors were stirred at room temperature for 24 h. Afterward, the composite photocatalysts were obtained by evaporating the ethanol. The STO content in the heterostructure varied from 20 to 50 wt %.

### 2.2 Heterostructure Characterization

Brunauer–Emmett–Teller (BET) surface area studies were carried out by N_2_ adsorption using a Micromeritics 2000 instrument.

X-ray diffraction (XRD) patterns were obtained using a Siemens D-501 diffractometer with a Ni filter and a graphite monochromator. The X-ray source was Cu Kα radiation. Refinement of unit cell parameters, anatase fraction, and crystallite size were performed by Rietveld fitting using HighScore-Plus software.

Micro-Raman measurements were performed using a LabRAM Jobin Yvon spectrometer equipped with a microscope. Laser radiation (λ = 532 nm) was used as an excitation source at 5 mW. All measurements were recorded under the same conditions (2 s of integration time and 30 accumulations) using a 100× magnification objective and a 125 mm pinhole.

Diffuse reflectance UV–vis spectroscopy was performed using a Cary 300 instrument. Spectra were recorded in the diffuse reflectance mode using Spectralon as a white standard. The scan range was 240–800 nm.

The transmission electron microscopy (TEM) images and high-angle annular dark field (HAADF) and elemental mapping analysis images were obtained by using a FEI Tecnai F30 microscope in the scanning transmission electron microscopy (STEM) mode operated at 300 kV equipped with a Gatan GIF Quantum 963 energy filter. The samples were directly dropped on a copper or nickel grid.

### 2.3 Photocatalytic Runs

**Figure 1:**
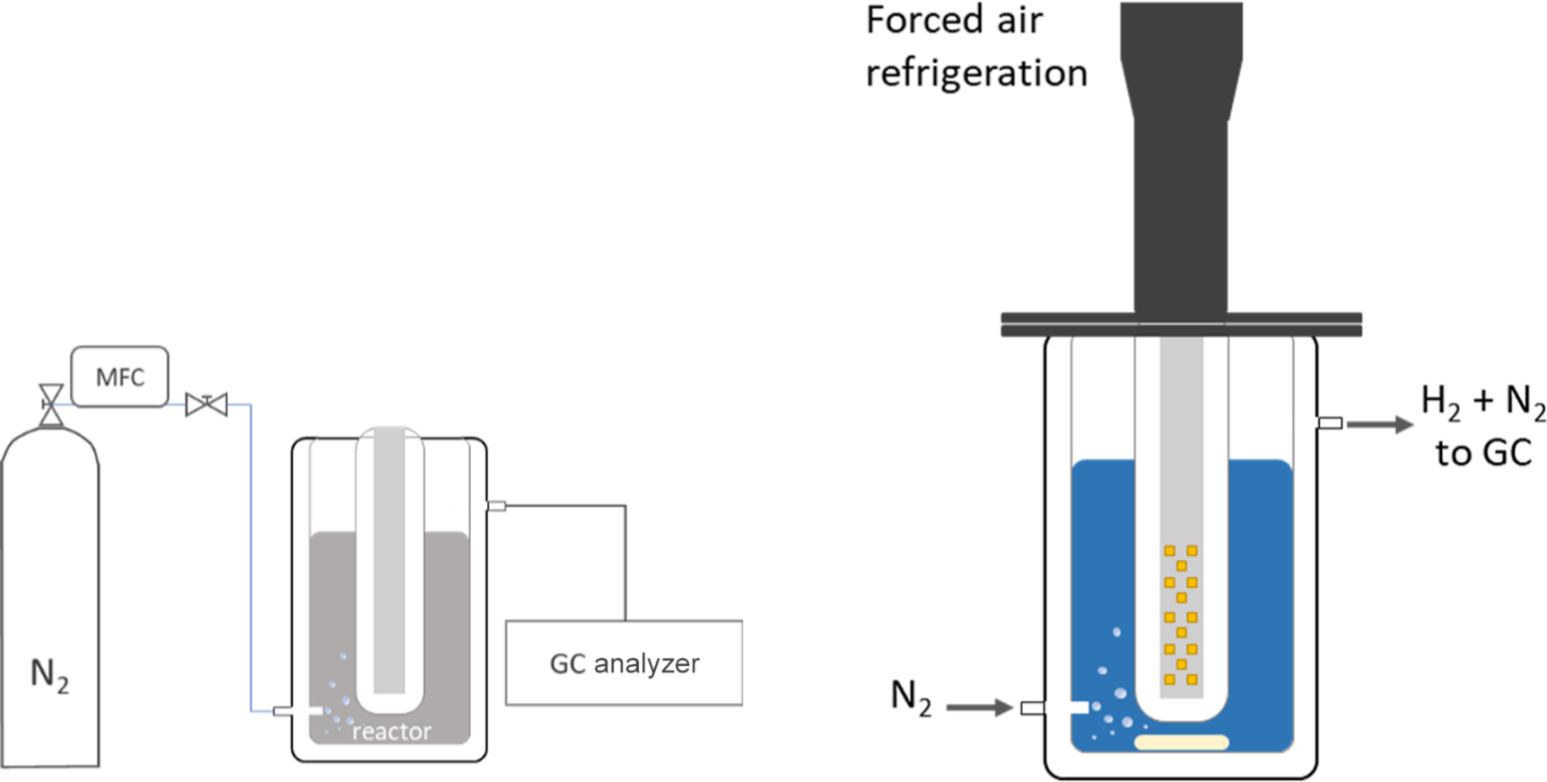
Flow scheme of the liquid-phase setup for catalytic testing (left side) and schematic drawing of the double-walled glass reactor with LED illumination (right side).

Photocatalytic H_2_ production tests were performed in a liquid-phase flow reactor system supplied by Apria Systems (Figure 1). The powder photocatalysts (0.5 g/L) were suspended in a water–methanol solution (10% vv) and then degassed with N_2_ at 50 mL/min for 60 min before the reaction. Then, the flow was settled at 20 mL/min, and the lamp (365 nm UV LED array) was switched on. The effluent gases were analyzed to quantify H_2_ production by gas chromatography (Agilent 7890B GC) using a thermal conductivity detector.

The apparent quantum yield (AQY) for the H_2_ evolution reaction was estimated from the reaction rate and the flux of incoming photons (calculated for the irradiation wavelengths of 365 nm) using the following equation.^35^
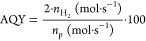
where  is the number of molecules of H_2_ generated and *n*_p_ is the number of incident photons reaching the catalyst. The number of incident photons has been calculated from the ratio between the total incident energy and the energy of a photon. In our experimental conditions, total incident energy was obtained from the wavelength of the incident light used (λ = 365 nm) and the power density of the incident light (2100 W m^–2^).

## 3 Results and Discussion

### 3.1 Structural and Surface Features

**Figure 2:**
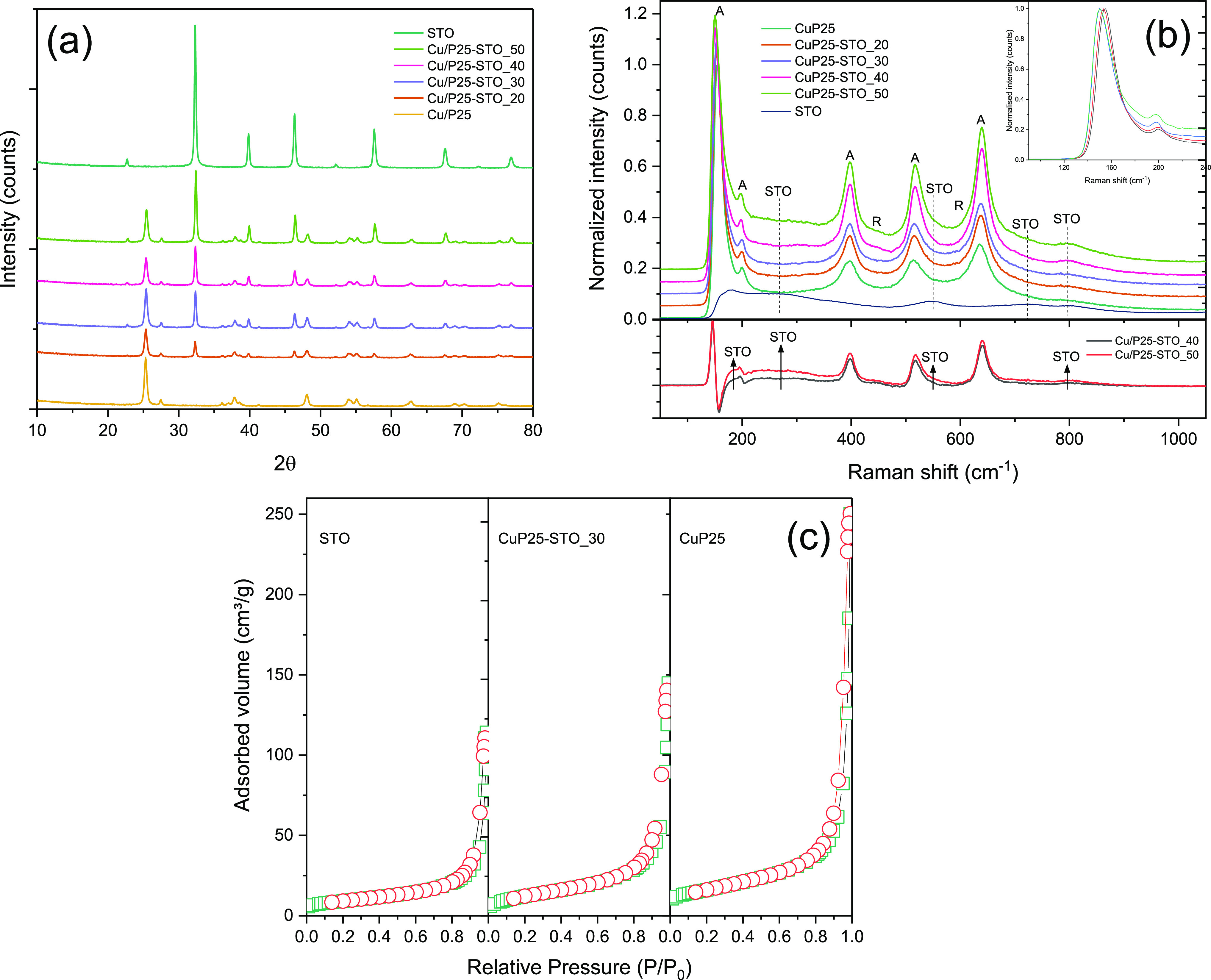
(a) XRD pattern; (b) Raman spectra (A: anatase; R: rutile; and STO: SrTiO_3_) and difference spectra with respect to CuP25; and (c) N_2_ adsorption–desorption isotherms for CuP25, CuP25–STO, and STO catalysts.

In Figure 2a, we show the XRD patterns of CuP25–STO heterostructured catalysts prepared by the simple co-deposition method. The XRD patterns of the SrTiO_3_ sample obtained by mw-assisted hydrothermal synthesis can be well indexed to the perovskite cubic phase (PDF 73-0661).^36^ No other peaks ascribed to other phases can be detected. On the other side, the CuP25 catalyst clearly shows the typical mixture of TiO_2_ anatase and rutile crystalline phases of commercial Evonik P25 TiO_2_. Finally, for CuP25–STO composites, it is possible to observe a lineal combination of phases detected for single systems. From Rietveld refinement, we obtained similar crystallite sizes for identified phases, being ca. 21, 32, and 44 nm for anatase, rutile, and SrTiO_3_, respectively.

In Table 1, we summarize the STO molar fraction calculated from Rietveld refinement. The evolution of STO content calculated from Rietveld refinement is linear along the whole range of studied series. We have also obtained the Raman spectra of studied systems (Figure 2b). As widely reported, the TiO_2_ anatase phase exhibits six Raman-active modes (A_1g_ + 2B_1g_ + 3E_g_) located at 150 (Eg_(1)_), 196 (Eg_(2)_), 396 (A_1g_/B_1g_), 516 (A_1g_), and 640 cm^–1^ (Eg_(3)_). Additionally, the TiO_2_ rutile phase shows five Raman active modes at 140 (B_1g_), 235 (multi-photon process), 445 (E_g_), 609 (A_1g_), and 825 cm^–1^ (B_2g_).^37^ Bands for the rutile phase can only be foreseen with difficult in all catalysts. The spectrum of SrTiO_3_ in Figure 2b agrees with that reported previously in the literature.^38^ The observed bands are attributable to O–Sr–O bending modes (TO2 mode located at 175 cm^–1^ and TO3 mode located between 246 and 346 cm^–1^), the Ti–O–Ti bending mode (between 610 and 750 cm^–1^), and the Ti–O mode (at 796 cm^–1^). For heterocomposite catalysts, the above-described bands for anatase predominate, and only for the CuP25–STO_50 catalyst can STO bands be hardly envisaged.

**Table 1:** Structural, Surface Features, and Calculated Band Gap Values for CuP25, STO, and CuP25–STO Systems

catalysts	% STO^a^	BET surface area (m^2^/g)	*E*_g_ (eV)
CuP25		57	3.10
CuP25–STO_20	13	52	3.10
CuP25–STO_30	22	47	3.11
CuP25–STO_40	31	46	3.11
CuP25–STO_50	41	44	3.12
STO	100	33	3.18

a Semi-quantitative STO phase molar fraction from Rietveld refinement.

By subtracting each normalized spectrum with that of CuP25, it is possible to see the increasing contribution of STO peaks specially for higher-STO-content heterocomposites (Figure 2b bottom panel). Additionally, it is possible to notice an overall increase in the peaks ascribed to the anatase phase. The intensity increases in the Raman peaks have been associated to the modification in the particle size, though such an assignment is certainly controversial.^39^

Moreover, the shift in the position to lower frequencies and the full width at half maximum broadening of the TiO_2_ anatase Raman lower energy E_g_ band (vibrational mode v5, assigned to the Ti/O bond stretching type vibrations) have been extensively discussed.^40^ Thus, the interpretations given about the variation of the peak position and shape of this E_g(1)_ Raman spectra involve different structural or morphological effects, which include particularly lattice parameter distortion and microstrain, non-homogeneous distribution of the particle size, and loss of stoichiometry due to oxygen deficiency. Such events can importantly contribute to the changes in the peak position, linewidth, and shape of the Raman mode in anatase TiO_2_ nanopowders. As seen in the Figure 2b inset, a progressive shift in the peak position can be envisaged as STO content increases. As previously argued, observed blue shift displacement can therefore be attributed to the close interaction of both crystalline phases, which would affect the structural features of TiO_2_. From these observations, we would anticipate that the composite would show certain heterostructuration and a close interaction between CuP25 and STO.

Surface areas calculated from N_2_ adsorption–desorption isotherms (Figure 2c) are summarized in Table 1. Preparation of STO by the mw-assisted hydrothermal method leads to a surface area of 33 m^2^/g, which is sensitively higher than the surface area of those prepared from solid-state reaction methods.^41,42^ In this sense, mw-assisted hydrothermal synthesis has been proposed as a convenient alternative in order to achieve high crystallinity without sacrificing high surface area values.^43,44^ Indeed, from the XRD pattern, a high crystallinity can be observed in our STO system. As expected, heterostructured CuP25–STO systems showed intermediate surface areas (Table 1). Thus, as STO content increases, the initial surface area exhibited by CuP25 (57 m^2^/g) progressively decreases, being 44 m^2^/g for 50 wt % STO content.

### 3.2 Morphological Studies of CuP25–STO Heterojunctions

**Figure 3:**
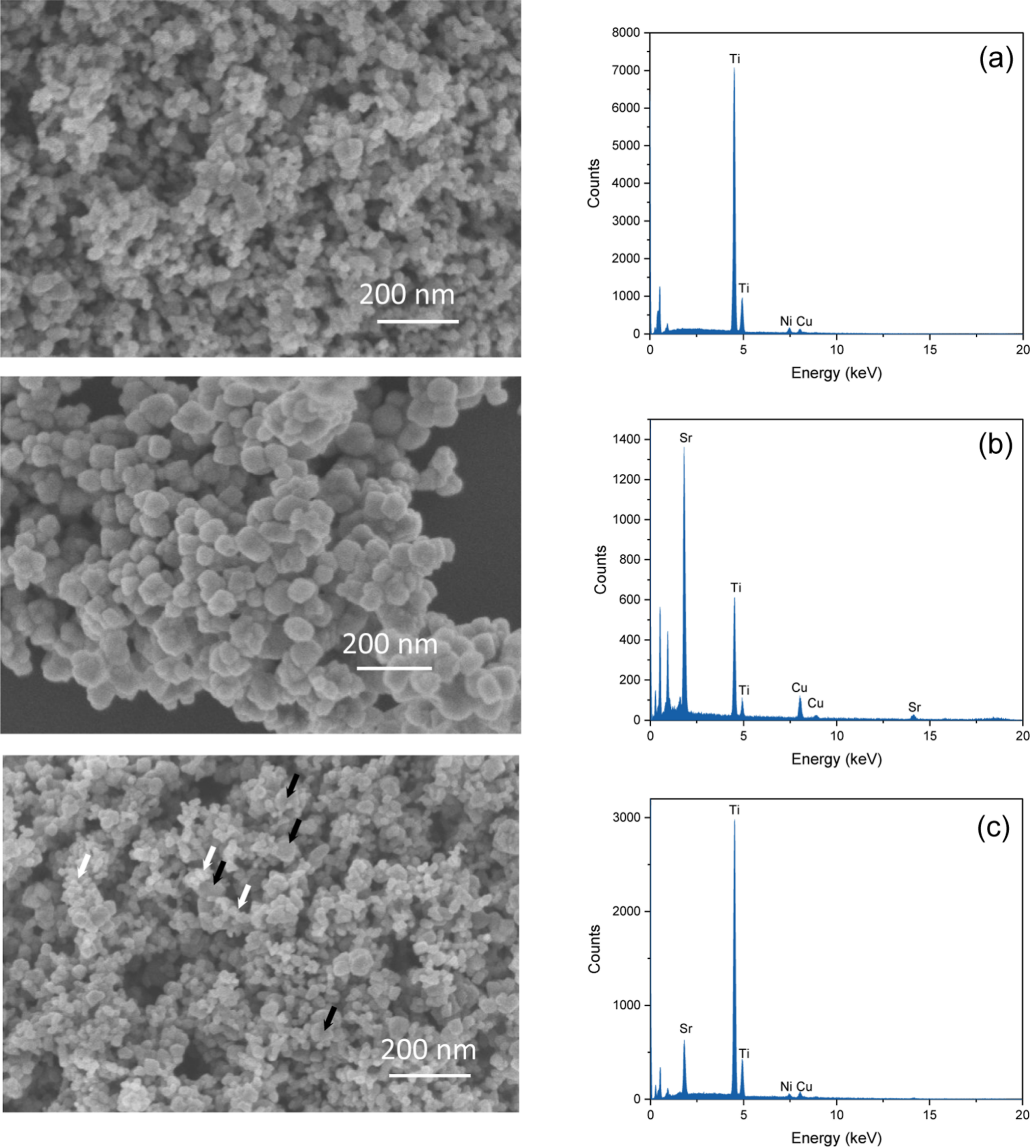
SEM images and energy-dispersive X-ray spectroscopy (EDX) analysis for (a) CuP25, (b) STO, and (c) CuP25–STO_30 catalysts (black arrows point out SrTiO_3_ particles and white arrows point out CuP25 particles).

The heterostructuration of the studied CuP25–STO systems has been assessed by electron microscopy. Thus, in Figure 3, we show scanning electron microscopy (SEM) images of former CuP25 and STO systems.

The CuP25 catalyst shows a very uniform distribution of rounded particles of ca. 20–30 nm size. On the other hand, the STO system presents slightly larger sizes of around 50–70 nm. In Figure 3, we also include the image of the CuP25–STO_30 system in which we can envisage both type of particles. It is also worth noting that higher particles, tentatively ascribed to STO, seem to be covered by the smaller ones, pointing out the pursued heterostructuration.

**Figure 4:**
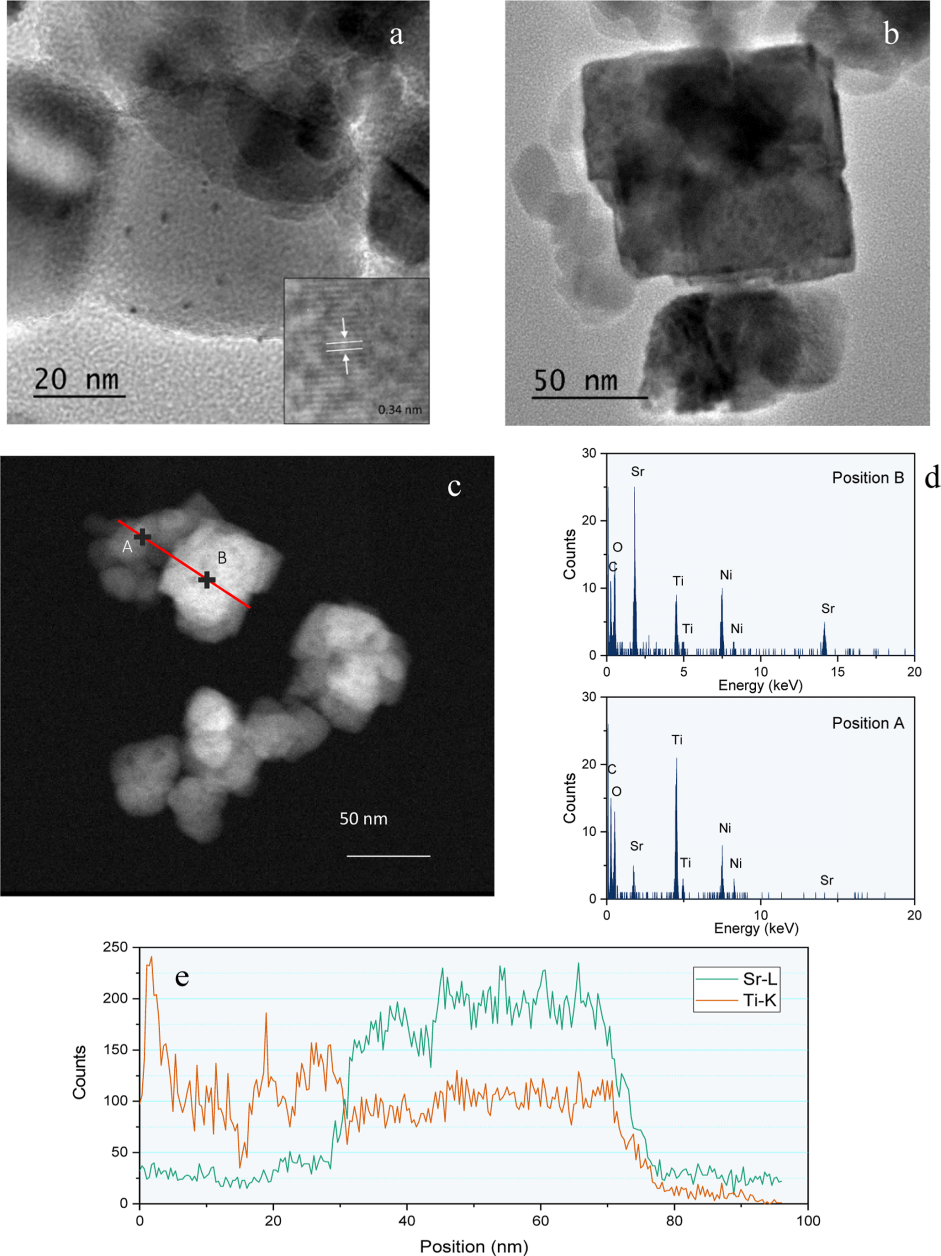
TEM images for (a) CuP25 and (b) CuP25–STO_30; (c) HAADF-STEM image for CuP25–STO_30; (d) local EDX analysis for A and B positions in Figure 6c; and (e) EDX line scan profiles for Sr and Ti across the red line in Figure 3c.

Such an intimate junction can be clearly observed from TEM images in Figure 4. The presence of well-dispersed Cu clusters over P25 can be clearly observed. The inset in Figure 4a clearly states that a Cu cluster is deposited over TiO_2_ anatase. The calculated plane distance corresponds to the (101) reflection from TiO_2_ anatase. These clusters showed a narrow distribution of size ca. 2–3 nm (Figure 4a). On the other hand and as previously discussed from the SEM image, STO in the CuP25–STO_30 catalyst exhibits a larger particle size, showing a cubic geometry resembling the cubic structure of the perovskite unit cell, being ca. 60 nm in size (Figure 4b). These nanocube-like particles appeared cleared surrounded by CuP25 smaller particles, as has been demonstrated by line profile analysis (Figure 4c).

### 3.3 Optical Properties

**Figure 5:**
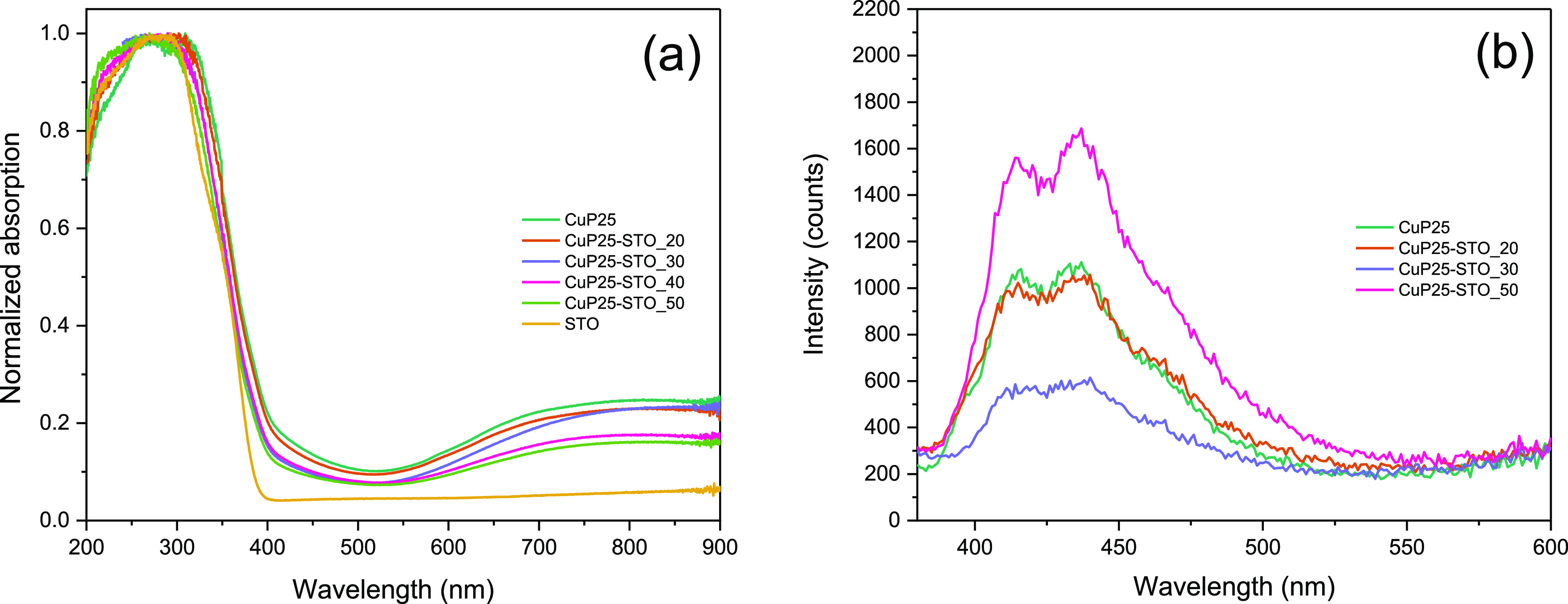
(a) UV–vis diffuse reflectance spectra for CuP25, CuP25–STO, and STO catalysts and (b) PL spectra for CuP25 and CuP25–STO catalysts after excitation at 350 nm.

Figure 5a shows the absorbance spectra of the CuP25–STO systems. The absorbance spectrum of STO extends from 200 to 400 nm and shows a band centered at around 320 nm, which corresponds to the absorption band edge of SrTiO_3_, leading to a band gap value of 3.18 eV (Table 1).^45^ With respect to CuP25, the UV–vis diffuse reflectance spectrum shows the typical absorption edge at around 350 nm, associated to the O^2–^(2p) → Ti^4+^(3d) charge transfer process, leading to a band gap value of 3.1 eV. The presence of Cu clusters at the surface produce an additional large absorption band between 450 and 800 nm.^46^ Heterostructured CuP25–STO systems show similar absorption profiles, but the mixed composition can be envisaged from the progressive increasing evolution of the band gap from CuP25 as STO content increases.

Photoluminescence (PL) spectra were also recorded for the heterostructured CuP25–STO catalysts (Figure 5b). Emission spectra in the range 380–650 nm were obtained upon excitation at 355 nm. The emission processes noticed on the PL curves are directly related to photogenerated charge carrier recombination.^47^ As can be seem from Figure 5b, the higher emission intensity would denote a higher recombination of the electron and holes. Thus, it is clear that the formation of the CuP25–STO heterojunction with 30 wt % of STO particularly improves the efficiency of charge pair separation with respect to that of single CuP25. The higher content of STO clearly favors the recombination of charge pairs, increasing the PL emission.

### 3.4 Photocatalytic H_2_ Production

**Figure 6:**
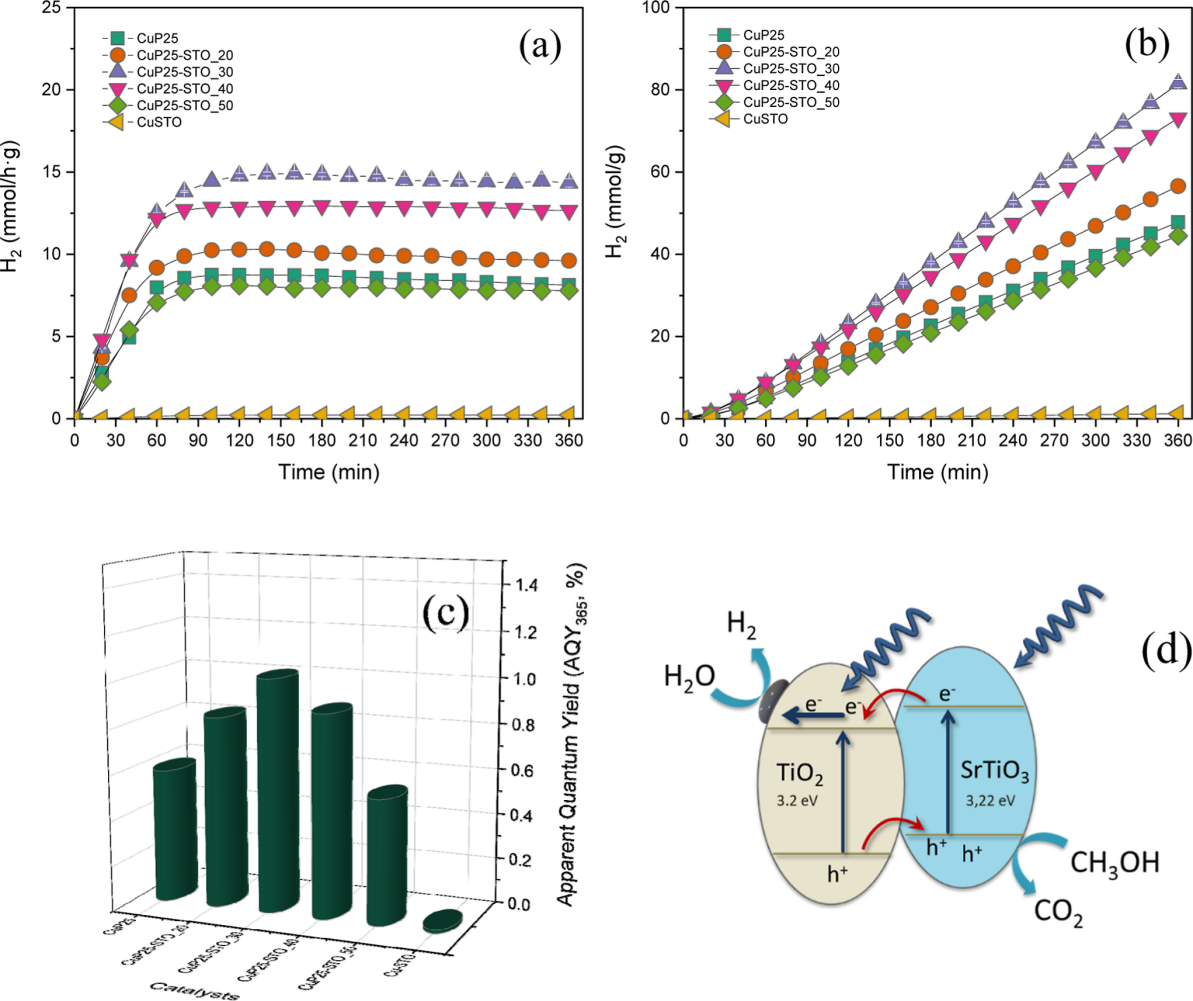
(a) H_2_ production rates; (b) H_2_ yields (we have included the error bars for CuP25–STO_30 catalyst); (c) calculated AQYs for CuP25, CuP25–STO, and STO catalysts; and (d) band scheme of heterostructured CuP25–STO systems.

The photocatalytic production of hydrogen has been studied by means of a continuous flow lab reactor. In Figure 6, we depict the H_2_ production rates and the yield evolution with time from methanol photoreforming. In the present study, we have obtained 8.7 mmol/h g of H_2_ production using the CuP25 catalyst. For the sake of comparison, we have included the photoactivity of CuSTO, which clearly shows a very low H_2_ production rate. The heterostructuration of CuP25 and STO at different STO contents leads to a significant increase in the H_2_ production up to 30 wt % of STO (Table 2).

**Table 2:** Rates for the H_2_ Photoreforming under Different Conditions

catalysts	H_2_ yield@6 h (mmol/g)
CuP25	47.8
CuP25–STO_20	58.6
CuP25–STO_30	81.7
CuP25–STO_40	73.1
CuP25–STO_50	44.5
CuSTO	1.2

For this catalyst, the accumulated H_2_ formed after 6 h of the reaction is 1.7 times higher than that with CuP25. After this content, the H_2_ formation rates suffers a progressive diminution. Thus, CuP25–STO_50 shows a similar photocatalytic activity to single CuP25. Calculated AQYs for the present reaction conditions are also shown in Figure 6. The obtained value for CuP25–STO_30 is ca. 1%.

The scheme for electron–hole separation (Figure 6d) and transport at the light-driven CuP25–STO hybrid photocatalyst can be discussed by considering the band structure of each semiconductor in the heterojunction. As reported in the literature, the conduction band (CB) and valence band (VB) edge potentials for STO were located at ca. −0.7 and 2.7 eV, respectively.^48,49^ Thus, the bottom (*E*_CB_) of the CB of SrTiO_3_ is located above that of TiO_2_ by ca. 0.35 eV.^50^ So, the photoexcited electrons on STO would be transferred easily to the TiO_2_ CB, while holes would be promoted toward the STO VB, leading to an effective charge carrier separation. In other words, CuP25 can act as a sink for photoexcited electrons, while STO would act as a sink for holes, hindering this way the charge recombination process, as has been inferred before from PL spectra (Figure 5).

## 4 Conclusions

We have obtained a highly active Cu/TiO_2_/SrTiO_3_ composite by a simple impregnation method. The obtained heterostructured Cu/TiO_2_/SrTiO_3_ composites show a significant improvement in the photocatalytic hydrogen production through methanol reforming. Such a marked increase in the H_2_ photoproduction might be related to an effective charge separation process. Thus, we have stated that photogenerated electrons at TiO_2_ and also those from SrTiO_3_ would directionally migrate to Cu/TiO_2_ due to the close interfacial connections between Cu/TiO_2_ and SrTiO_3_. This would lead to a significantly lower recombination of the charges. Thus, this driving apart would retard the charge recombination and improve the photoactivity for H_2_ production. As a result, the higher efficiency achieved in the electronic step is responsible for the enhanced photocatalytic hydrogen production reactions. The best photoproduction is attained for the 30 wt % SrTiO_3_ heterojunction showing 81.7 mmol/g H_2_ after 6 h, 1.7 times higher than that of bare Cu/TiO_2_. So, by the adequate tailoring of the catalysts, it is possible to optimize the charge carriers handling and hinder the recombination process.
